# A Deep Learning Prediction Model for Structural Deformation Based on Temporal Convolutional Networks

**DOI:** 10.1155/2021/8829639

**Published:** 2021-04-20

**Authors:** Xianglong Luo, Wenjuan Gan, Lixin Wang, Yonghong Chen, Enlin Ma

**Affiliations:** ^1^School of Information and Engineering, Chang'an University, Xi'an 710064, China; ^2^China Railway First Survey and Design Institute Group Co., Ltd., Xi'an 710043, China; ^3^School of Highway, Chang'an University, Xi'an 710064, China

## Abstract

The structural engineering is subject to various subjective and objective factors, the deformation is usually inevitable, the deformation monitoring data usually are nonstationary and nonlinear, and the deformation prediction is a difficult problem in the field of structural monitoring. Aiming at the problems of the traditional structural deformation prediction methods, a structural deformation prediction model is proposed based on temporal convolutional networks (TCNs) in this study. The proposed model uses a one-dimensional dilated causal convolution to reduce the model parameters, expand the receptive field, and prevent future information leakage. By obtaining the long-term memory of time series, the internal time characteristics of structural deformation data can be effectively mined. The network hyperparameters of the TCN model are optimized by the orthogonal experiment, which determines the optimal combination of model parameters. The experimental results show that the predicted values of the proposed model are highly consistent with the actual monitored values. The average RMSE, MAPE, and MAE with the optimized model parameters reduce 44.15%, 82.03%, and 66.48%, respectively, and the average running time is reduced by 45.41% compared with the results without optimization parameters. The average RMSE, MAE, and MAPE reduce by 26.88%, 62.16%, and 40.83%, respectively, compared with WNN, DBN-SVR, GRU, and LSTM models.

## 1. Introduction

With the rapid development of social economy, the demand is continuously growing for large structural engineering such as subways, bridges, and tunnels, and their safety is also becoming more and more important in the stage of constructions and operations. Since the structural engineering is subject to various subjective and objective factors, the deformation is usually inevitable. Serious deformation even causes disaster accidents, which have brought huge losses to people's lives and property safety. Therefore, safety requirements are imposed during the construction and operation stages, and the automatic monitoring system of structural deformation has become indispensable. The structural deformation prediction model can grasp the deformation trend using monitoring data, so that emergency measures can be taken to prevent the occurrence of disasters in advance. Therefore, accurate and real-time deformation prediction model has also become a research hotspot in the structural automation monitoring system.

Many structural deformation prediction models had been proposed in the past few years. The existing methods mainly include the regression analysis, gray theory, time series analysis, neural network, and combined prediction models. There are many factors that cause structural deformation, such as monitoring equipment, structural geological conditions, physical properties, and other external environment. The different prediction models have their advantages and adaptability for different monitoring data. Regression analysis is a mathematical statistical method by determining the relationship between structural deformation and relevant factors. The regression method is very effective for fewer variables and data with obvious regularity [1]. It is often used in dams [2, 3] and slopes [4–7] deformation prediction. Dai et al. [8] studied the concrete dam deformation prediction model based on statistical and random forest regression (RFR). The RFR method was used to extract representative influence factors according to the different importance, and then, the important factors were used to describe the concrete dam deformation. The results showed that the RFR model was very effective to analyze and predict the dam deformation. The regression analysis model is a kind of the static model, which cannot fully consider the internal correlation characteristics of structural monitoring data. At the same time, some deformation factors are not measurable [9], which will also affect the accuracy of the prediction model. In 1982, Deng [10] proposed the gray system theory (GST), and the gray prediction model based on the nonstatistical method is one of the significant contents of the gray theory. It has better prediction performance for complex systems with uncertain factors and smaller sample data. Based on the traditional gray GM (1, 1) model, Zhu [11] used the gray-fuzzy Markov chain to predict landslide deformation, and the prediction accuracy was improved by optimizing the prediction error. Aiming at the shortages of equal interval models, Qing [12] established a multivariate gray model with unequal spacing to predict soil creep deformation, and the creep deformation range can be predicted effectively. However, the gray system requires that the data sequence has nonnegative characteristics, and the data accumulation has a gray-index law, which results in that the extensive application of the gray theory is limited in structural deformation prediction.

Since the structural deformation data are a typical time series, the typical time series analysis methods including the autoregressive moving average (ARMA) [13, 14] and its improved method autoregressive differential moving average (ARIMA) [15, 16] models have been used to predict structural deformation. Chen et al. [14] established a deformation prediction model based on ARMA, and the analysis results of the real measured data showed that this method had high reliability and accuracy. Xu et al. [16] utilized ARIMA and ARMA to predict structural deformation, which can accurately reflect the buildings deformation by the analysis of time domain characteristics. The time series analysis requires the data to be linear and stationary; however, the structural deformation monitoring data in engineering have complex and nonlinear characteristics, which will affect the prediction accuracy.

In recent years, neural networks and their improved algorithms have been successfully applied in structural deformation prediction [17–24]. Luo et al. [17] proposed a soft soil foundation settlement deformation prediction model with the TS fuzzy neural network, but the proposed method is only valid for short-term settlement. Kao and Loh [23] used artificial neural network methods to extract dam deformation features and predict long-term dam static deformation. However, the prediction results of neural networks are greatly influenced by the experimental data and model parameters, and it is also easy to fall into local optimum.

Because there are many factors that need to be considered, it is difficult for a single model to achieve desired accuracy. Therefore, aiming at the problems of the single model, the combined prediction models were often used to predict structural deformation [2, 25–29] or classify [30, 31]. Jiang et al. [26] proposed a least square support vector machine-Markov chain (LSSVM-MC) model to improve dam prediction accuracy. Chen et al. [27] established a safety monitoring model for earth dams with the radial basis neural network (RBF-NN) and kernel principal component analysis (KPCA). Xin et al. [28] established the Kalman-ARIMA-GARCH (generalized autoregressive conditional heteroskedasticity) model to improve bridge structure deformation prediction accuracy. Luo et al. [29] proposed a prediction model of structural settlement based on EMD-SVR-WNN. The influence of different components on the prediction can be considered, and the experimental results show that the proposed model is an effective model of structural settlement. The combined prediction models combine the advantages of different models, but the predictive performance is greatly affected by the fusion algorithm.

With the development of information technology, deformation monitoring data are entering the era of big data. It is also possible to predict structural deformation using artificial intelligence methods, especially deep learning. At present, the deep learning method is gradually applied to structural deformation prediction. Yang et al. [32] used the LSTM (long short-term memory) model to predict the landslide periodic displacement. The results showed that the LSTM model could make full use of historical information to improve the model performance. Qiu et al. [33] proposed the Levenberg–Marquardt and conditional deep belief network (LM-CDBN) model to predict the supertall buildings deformation. The results indicated that the optimization algorithm had high prediction accuracy. Pu et al. [25] utilized the dynamic linear model and long short-term memory (DLM-LSTM) model to predict land deformation. Li et al. [34] utilized LSTM to predict landslide susceptibility through landslide instability factors, the results indicated that LSTM outperformed the other three models (decision tree (DT), support vector machines (SVM), and the back propagation neural network (BPNN)) because of its capability to learn time series with long temporal dependencies. Although LSTM can obtain long-term memory of the time sequences, it is not widely applied due to many parameters and long training time.

The TCN (temporal convolutional network) is an improved CNN algorithm, and its applicability was proved by Bai et al. [35]. The TCN can overcome the shortcomings of LSTM in solving time series; at meanwhile, the TCN has strong feature extraction ability and simpler structures, which provide a new method for structural deformation prediction. According to the characteristics of the TCN model, a structural deformation prediction model was proposed based on the TCN in this study. First, for the next step of data analysis to seek the optimal solution and improve the accuracy, the data are preprocessed and normalized and then divided into training set and test set. Second, according to the proposed TCN model, the time feature is extracted, and finally, the prediction output is obtained from the full connection layer by calculating the prediction errors. By changing the dilated factor flexibly, long history information of monitoring data can be obtained, and data internal characteristics can be fully mined. At the same time, the causal convolution and residual connection can prevent future information leakage and effectively improve the prediction accuracy, respectively. Third, orthogonal experimental design is adopted to optimize the hyperparameters of the TCN model. Although the TCN model also has simpler structure than traditional recursive networks, the superparameters also affect not only the calculation speed but also final prediction accuracy the same as typical deep learning models. The optimal hyperparameters combination of the model can be found within a certain range. The model was verified by the measured data, and the results indicated that the proposed method has lower prediction error and higher accuracy compared with the existing prediction models, and it is an effective structural deformation prediction model, which can provide important decision support for structural safety prevention.

The rest of this study is organized as follows.  Section 2 introduces the basic theory of the TCN and the proposed structural deformation prediction model based on the TCN in detail. In Section 3, the dataset used is introduced for the numerical experiments. The results and performance evaluation are presented. The hyperparameters of the TCN model are also analyzed. Finally, the conclusions and the future research are given in Section 4.

## 2. Temporal Convolutional Sequence Model

The TCN is a structural innovation of the one-dimensional CNN. It adds dilation factors in the traditional convolution, which can cover all historical information, effectively use historical information to solve timing problems, and add causal convolution to prevent future information leakage. The TCN model is widely used in speech recognition and time series because of its simple structure and flexible receptive field. Since structural deformation data are a typical time series, the TCN is used to establish the deformation prediction model in this study.

### 2.1. Causal Convolutions

**Figure 1:**
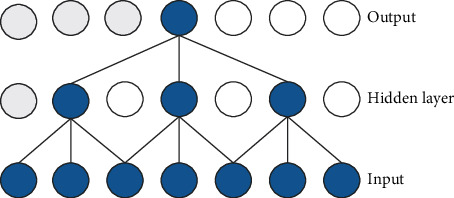
Noncausal convolution kernel size (*k* = 3).

**Figure 2:**
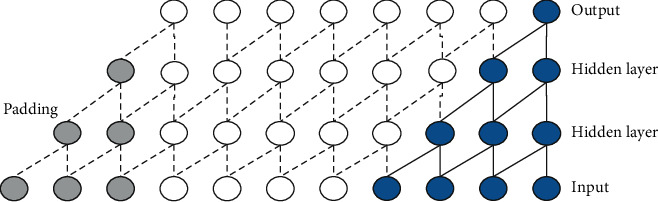
Causal convolution kernel size (*k* = 2).

The TCN model mainly has two principles, the output sequence of the network should have the same length as the input sequence, and the network can only use the information from past time steps. According to the first principle, the TCN uses the 1D full-convolutional network to ensure that all convolution layers have the same length after zero padding. According to the second principle, the TCN uses causal convolutions, which means that output *y*_*t*_ depends on the input {*x*_0_, *x*_1_, *x*_2_,…, *x*_*t*_} but is independent of {*x*_*t*+1_, *x*_*t*+2_, *x*_*t*+3_,…, *x*_*t*+*T*_}. Noncausal and causal convolutions are shown in Figures 1 and 2.

The output of causal convolutions in the time step *t* will only be derived from the convolution operation at time *t* and before, ensuring that the prediction of the previous time step does not use future information. Causal convolution is shown in the following equation.

(1) $$\begin{array}{c} p\left(x\right)=\prod_{t=1}^{T}p\left(x_{t}|x_{0},\ldots ,x_{t-1}\right). \end{array}$$

In order to obtain a long-term memory of sequence, causal convolutions need to increase convolution kernel or deepen the network depth, which would lead to an increase of calculation.

### 2.2. Dilated Convolutions

Since the structural deformation data have a strong temporal correlation characteristic, the analysis of structural deformation not only considers the data in previous moment but also in a long time ago. Generally, there are usually three methods to broaden the receptive field with a simple causal convolution. The first method is to deepen the network depth. However, with the network deepening, the network training is complicated and the fitting effect is not necessarily well. The second method is to increase the size of the network convolution kernel. With the increase of convolution kernel size, the receptive field increases linearly. This method enlarges the receptive field, but increases the parameters and network complexity. In general, multiple small overlaying convolution kernels can greatly reduce the number of parameters and computational complexity compared with a large convolution kernel separately. The third method is to increase the step. When the convolution step size is too large, the output sequence length will be reduced due to downsampling. In addition, it is possible to omit the effective information and affect the accuracy of feature extraction.

In order to overcome these shortcomings, Yu and Koltun [36] proposed the dilated convolutions and established a multiscale context aggregation architecture based on dilated convolution, which can exponentially enlarge receptive field without limiting coverage. Zhang et al. [37] proposed multiscale single image superresolution (SR) based on dilated convolutions, and good performances were achieved because the dilated convolution broaden the receptive field without increasing the parameters and reducing the resolution.

**Figure 3:**
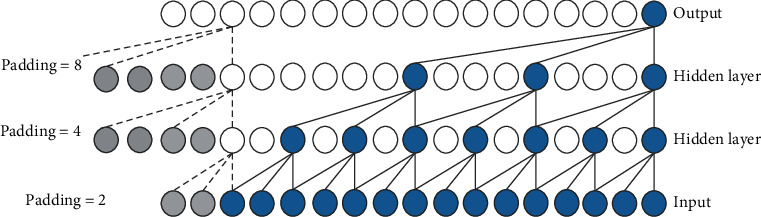
Causal dilated convolution (*k* = 3).

By skipping the input value with the given step size, the dilated convolution obtains the long-term memory of the sequence without increasing the calculation amount. Formally, for a one-dimensional time sequence input *X*={*x*_1_, *x*_2_,…, *x*_*N*_} and a convolutions kernel *F*={0,1,…, *k* − 1}, the dilated convolution is defined in the following equation.

(2) $$\begin{array}{c} \bar{F}\left(s\right)=\left(X{*}_{d}F\right)\left(s\right)=\sum_{i=0}^{k-1}F_{i}*X_{s-d\cdot i}, \end{array}$$

where *d* is the dilated factor, *k* is the convolution kernel size, and *s* − *d* · *i* accounts for the direction of the past. Generally, the dilated factor *d* increases exponentially with the increase of network depth (*d*=2^*i*^, *i* is the number of layers in the network), which ensures that the network can cover all valid historical information. Dilated convolution becomes regular convolution when *d*=1. An illustration of causal dilated convolution with the dilated factor *d*={1,2,4,8} is shown in Figure 3, and it is obvious that the receptive field increases with the increase of the layer-by-layer dilated factor.

### 2.3. Residual Connection

The receptive field of the TCN depends on the convolution kernel size *k*, the dilation factor *d*, and the depth *n* of the network. As the network deepening, the parameters of the network model increase, which will lead to gradient dispersion or gradient explosion, and the accuracy of the training set will be saturated or even degraded. In order to solve these problems, the residual connections are added in the TCN, which can optimize the deep network, simplify the training process, and significantly improve network performance. The residual connections are defined in the following equation.

(3) $$\begin{array}{c} O=\text{Activation}\left(X+F\left(x\right)\right), \end{array}$$

where *X* is the convolution input, *F*(*x*) is the convolution output, and *O* is the output of the residual connections. In this study, the nonlinear activation function is used, which is defined in the following equation.

(4) $$\begin{array}{c} f_{\text{ReLu}}=\max\left(0,X_{i}\right). \end{array}$$

The ReLu function can maintain the gradient without attenuation at *X*_*i*_ > 0, which can alleviate the gradient disappearance problem, achieve the same training performances, and operate faster than the traditional activation functions, such as sigmoid and tanh.

**Figure 4:**
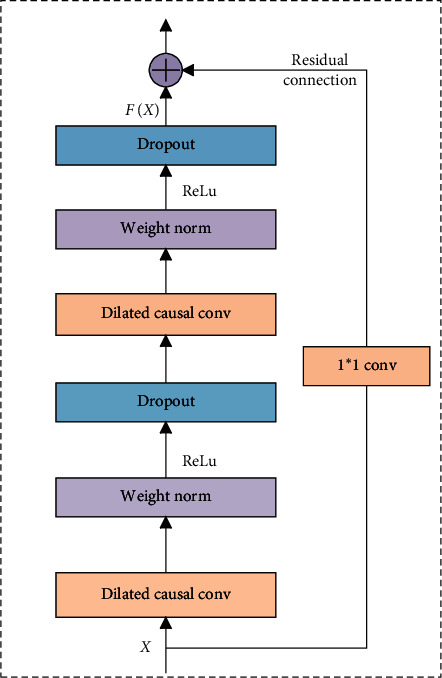
Residual connections in the TCN.

The residual module of the TCN is shown in Figure 4, and there are two layers of dilated causal convolution and ReLu in each residual module. After each dilated causal convolution, the weight normalization layer and the dropout layer are added to improve the network generalization. Finally, when the residual input and output have different dimensions, a 1 × 1 convolution is added to ensure that the input and output have the same dimensions. The residual connections can effectively improve the ability of feature extraction and network stability in deep networks.

### 2.4. Structural Deformation Prediction Model

**Figure 5:**
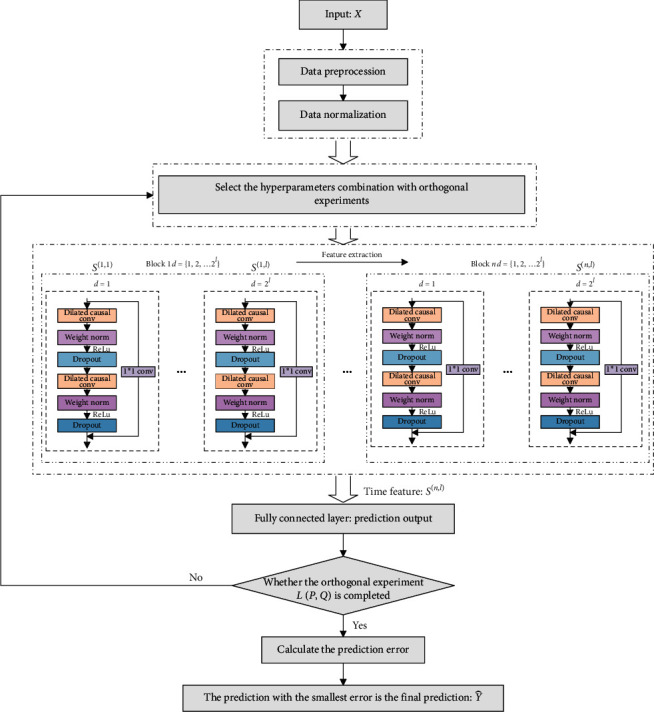
The flowchart of the proposed prediction model.

In order to ensure the safety of the structure, the application of monitoring equipment is wide and essential, and the structural deformation monitoring data are obtained by the automatic monitoring equipment in a certain sampling interval, and it is a typical time sequence which reflects the rules of structural health changing in the time field. The future structural deformation trend can be predicted by analyzing the monitoring historical data [38]. Combined with the advantages of the TCN, a structural deformation prediction model is proposed based on the TCN in this study, and the flowchart of the proposed method is shown in Figure 5.

As shown in Figure 5, a series of blocks are defined in the model. The dilation factor in each block increases exponentially (*d*=2^*i*^, *i* is the number of layers of convolution). The output in the *i*^th^ (1 ≤ *i* ≤ *n*) block and *j*^th^ (1 ≤ *j* ≤ *l*) layer is defined as *S*^(*i*, *j*)^ ∈ ℝ^*Fw*×*N*^, and the input of each block *S*^(*i*, 1)^ comes from the previous block *S*^(*i* − 1, *l*)^, except the input of the first block, which is the input dataset *X*. The depth network is composed to fully extract time features.

(5) $$\begin{array}{c} S_{t}^{\left(i,j\right)}=\mathrm{ReLu}\left(S_{t}^{\left(i,j-1\right)}+{\bar{S}}_{t}^{\left(i,j\right)}\right)\text{,}\;1\leq i\leq n,\;1\leq j\leq l, \end{array}$$

where *S*_t_^(*i*, *j*)^ denotes the results of each layer residual connection, and ${\bar{S}}_{\text{t}}^{\left(i,j\right)}$ is the result of each layer dilated convolution at time *t*.

(6) $$\begin{array}{c} {\hat{Y}}_{t}=g\left(W\cdot S_{t}^{\left(n,l\right)}+b\right). \end{array}$$

In equation (6), *W* and *b* are the parameters obtained by training, *S*_*t*_^(*n*, *l*)^ is the output of the time characteristics, and *g*(·) is the activation function. In this study, the activation function uses the linear activation function, and the output is predicted by full connection.

(7) $$\begin{array}{c} X_{tr}={\left[\begin{array}{ccccc} x_{1} & x_{2} & x_{3} & \ldots & x_{L}\\ x_{2} & x_{3} & x_{4} & \ldots & x_{L+1}\\ \ldots & \ldots & \ldots & \ldots & \ldots \\ x_{M-L} & x_{M-L+1} & x_{M-L+2} & \ldots & x_{M-1} \end{array}\right]}_{\left(M-L\right)\times L}, \end{array}$$

(8) $$\begin{array}{c} Y_{tr}={\left[\begin{array}{ccccc} x_{L+1} & x_{L+2} & x_{L+3} & \ldots & x_{M} \end{array}\right]}_{T}^{1\times \left(M-L\right)}. \end{array}$$

Suppose that *X*=[*x*_1_, *x*_2_, ⋯,*x*_*i*_, ⋯, *x*_*N*_] is the structural data monitored by a single sensor, *N* is the number of sampling points, and *L* is the length of the sliding window. The training input and output sets for the TCN network and the test input set are determined according to the structural deformation data. The top *M* sample points are used as the training set, the latter *N* − *M* sample points are used as the prediction set, and the training input and output sets are specified as in equations (7) and (8).

(9) $$\begin{array}{c} {\hat{Y}}_{tr}=\Phi \left(X_{tr},\theta \right). \end{array}$$

In equation (9), *X*_*tr*_ is the training input, and *Y*_*tr*_ is the target output. Φ(·) is the TCN model function, *θ* is the hyperparameter in the TCN model, and ${\hat{Y}}_{tr}$ is the training output. First, all parameters in the model are initialized. Then, the time feature extracted through the TCN layer is used as the input of the fully connected layer, and the training output ${\hat{Y}}_{tr}$ is finally obtained through the linear relationship. Then, the optimal solution of model parameter is found by minimizing the loss function.

Suppose that there are *P* hyperparameters in the TCN model and each hyperparameter has *Q* values, then the suitable orthogonal experiment *L*(*P*, *Q*) can be designed. Through the analysis of different hyperparameters and their values, the hyperparameter combination with the smallest error is finally selected as the optimized values according to the following equation.

(10) $$\begin{array}{c} E\left({\hat{\theta }}_{\text{opt}}\right)=\underset{\theta_{i}\in \Theta }{\min}\left\{E\left(\theta_{i}\right)\right\}, \end{array}$$

where *E* represents the prediction error, ${\hat{\theta }}_{\text{opt}}$ means the optimal hyperparameters combination, and Θ stands for the set of superparameter orthogonal experiment combinations.

(11) $$\begin{array}{c} X_{T}={\left[\begin{array}{ccccc} x_{M-L+1} & x_{M-L+2} & x_{M-L+3} & \ldots & x_{M}\\ x_{M-L+2} & x_{M-L+3} & x_{M-L+4} & \ldots & x_{M+1}\\ \ldots & \ldots & \ldots & \ldots & \ldots \\ x_{N-L} & x_{N-L+1} & x_{N-L+2} & \ldots & x_{N-1} \end{array}\right]}_{\left(N-M\right)\times L}. \end{array}$$

In order to verify the validity of the model, *X*_*T*_ is the input into the trained network, and the predicted output is obtained by the following equation with the optimized parameters determined through orthogonal experiments.

(12) $$\begin{array}{c} {\hat{Y}}_{T}=\Phi \left(X_{T},{\hat{\theta }}_{\text{opt}}\right). \end{array}$$

The specific steps for the proposed method are as follows:

  Step 1: the original data are preprocessed and normalized, and the training and test sets are divided.
  Step 2: the hyperparameters of the TCN model are selected through orthogonal experiments
  Step 3: the structural deformation prediction result can be obtained by the TCN model
  Step 4: calculate the prediction errors
  Step 5: repeat steps 1–4 with all hyperparameters combinations
  Step 6: obtain the predicted structural deformation when the error is the smallest

## 3. Data and Data Preprocessing

**Figure 6:**
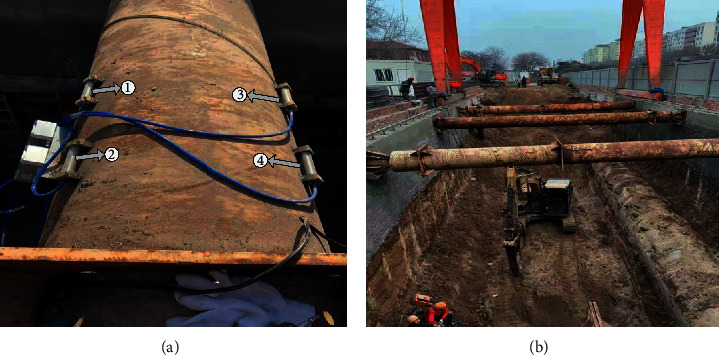
The project site for data acquisition.

In order to verify the availability of the proposed model, the experiments are executed with the cumulative strain data of the upper steel beam in a foundation pit in China. As shown in Figure 6, the structural subsidence data at the same location are monitored by four sensors. Regardless of data correlation, the data monitored by one of the sensors are only used for analysis. The monitoring data are collected from August 7th, 2019 to September 22nd, 2019, with a total of 1376 points. Due to the complex monitoring environment, data anomalies may be caused during data collection, transmission, and storages. In order to ensure data reliability, the data anomalies are eliminated at first.

In order to accelerate the speed of gradient descent, seek the optimal solution, and improve the accuracy, the training samples are normalized according to equation (13). Because the validation set is usually used to adjust the hyperparameters and orthogonal experiments have been used to optimize the hyperparameters, the validation set is not used in our experiment. The dataset is only divided into training and test set in this study. The first 70% of the data is used as a training set, and the last 30% is used as a test set.

(13) $$\begin{array}{c} {\tilde{X}}_{i}=\frac{\left(X_{i}-X_{\min}\right)}{\left(X_{\max}-X_{\min}\right)}\text{,}\;1\leq i\leq N. \end{array}$$

In equation (13), *X*_*i*_ represents the original settlement data, *X*_min_ represents the minimum value of the original sequence, *X*_max_ represents the maximum value of the original sequence, and ${\tilde{X}}_{i}$ represents the normalized data.

**Figure 7:**
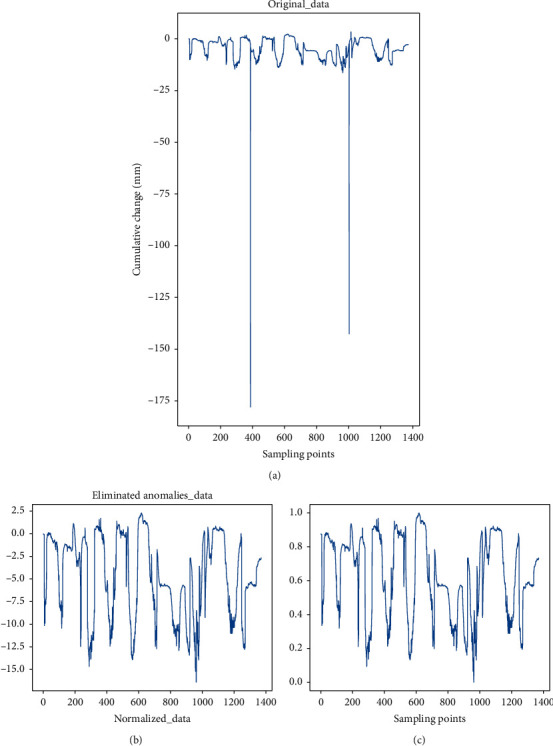
The original and preprocessed data.

The original and preprocessed data are shown in Figure 7. It can be seen that the original data contain a small number of abnormal points, and the data after the removal of abnormal samples can more effectively reflect the deformation trend of the foundation pit. The trend of the preprocessed data is highly consistent and matched with the original data even at the inflection point.

## 4. Experimental Results and Performance Analysis

### 4.1. Model Evaluation Index

In order to verify the validity of the model, some models [39–42] use statistical testing methods to ensure the superiority of the model. Common indicators used to evaluate the accuracy of prediction models in structural deformation prediction models include mean square error (MSE), root mean square error (RMSE) [43], mean absolute error (MAE), and mean absolute percentage error (MAPE). RMSE is very sensitive to large or small errors and can well reflect the measurement precision. MAE can avoid the problem of offset between deviations. MAPE not only considers the error between the predicted and true value but also considers the ratio between the error and the true value. In this experiment, RMSE, MAE, and MAPE are selected as the evaluation indicators. The smaller the values of RMSE, MAE, and MAPE, the higher the prediction accuracy of the model is. The calculation formula is as follows:

(14) $$\begin{array}{c} \text{RMSE}=\sqrt{\frac{1}{N}\overset{N}{\underset{i=1}{\sum }}{\left(y_{i}-{\hat{y}}_{i}\right)}^{2}}, \end{array}$$

(15) $$\begin{array}{c} \text{MAE}=\frac{1}{N}\overset{N}{\underset{i=1}{\sum }}\left|{\hat{y}}_{i}-y_{i}\right|, \end{array}$$

(16) $$\begin{array}{c} \text{MAPE}=\frac{1}{N}\overset{N}{\underset{i=1}{\sum }}\left|\frac{{\hat{y}}_{i}-y_{i}}{y_{i}}\right|\times 100\%. \end{array}$$

In equations (14)–(16), *y*_*i*_is the actual observed value of the deformation data, ${\hat{y}}_{i}$ is the predicted value, and *N* is the number of test series.

### 4.2. Model Hyperparameter Optimization

Since the proposed model is a deep learning model, network parameters have an important influence on the experimental results. In order to discuss the influence of hyperparameters on the model performance, the optimized model is found by analyzing parameter combinations that may affect prediction performance. The orthogonal test is an experimental method to study multifactors and multilevels through orthogonal tables. It is based on the principles of uniformity and orthogonality. By selecting factors that have a greater influence on the test results, partial experiments can effectively replace comprehensive experiments. It has the advantages of high efficiency and precision to find the optimal parameter combination. Some key hyperparameters *θ* are discussed in this study. The factors of orthogonal experiment are set as convolution kernel size, convolution kernel numbers, dilation factor, TCN layer numbers, and learning rate of the TCN prediction model, which are expressed by A, B, C, D, and E, respectively. The level of each factor is set as 4 according to existing experience, which are expressed by numbers 1, 2, 3, and 4, respectively. The specific parameter setting is given in Table 1.

In order to verify the effectiveness and stability of the proposed model, each experiment is carried out five times, and the average value was taken as the final result. The computer configuration for the experiment is given in Table 2.

Under the experimental environment and the parameters conditions in this study, the test results of the model hyperparameters optimization experiments are given in Table 3 according to the orthogonal test table. By analyzing the RMSE, MAPE, MAE, and running time of the different prediction results, the hyperparameters combination with the best model performance is selected based on the principle of minimum prediction error, highest accuracy, and relatively short running time.

From the experimental results in Table 3, it can be seen that the prediction errors in experiment number 14 are the smallest and the running time is relatively short. Since the structural deformation data are a long-term sequence with temporal correlation characteristic, as the increase of dilated factors and convolution kernels numbers in the causal dilated convolution, the output at the current moment *t* can be obtained, which is not only related to the input at the current moment but also the long-term input in the past. Temporal features can be fully extracted from the long-term memory of the time series. This method enlarges the receptive field without increasing the parameters and network complexity. But with the increase in the number of TCN layers, the temporal characteristics of the sequence can be fully mined, it is difficult to train the network parameters, which leads to longer training time, and the superparameters are more difficult to search. Orthogonal experiments avoid the shortcomings of the traditional optimization algorithm with too many parameters, and it can ensure the prediction accuracy of the results to the greatest extent.

The parameters of the TCN prediction model are set as follows: the size of the convolution kernel is 8, the number of convolution kernels is 16, the dilation factor is set as 32, the learning rate is 0.05, the number of TCN layers is 8, residual connections are adopted between TCN layers, the optimization function of the model is Adam, and the loss function is chosen as RMSE. The average RMSE, MAPE, and MAE of the optimized model parameters reduce 44.15%, 82.03%, and 66.48% respectively, and the average running time is reduced by 45.41% at the same time.

**Figure 8:**
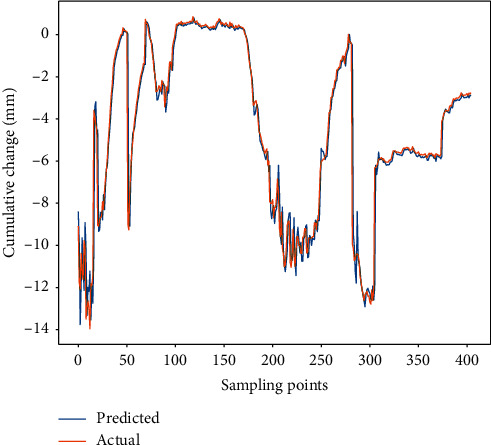
The prediction results of the proposed model.

The predicted results and the actual measured values are shown in Figure 8. The results show that the predicted values are very close to the measured values. Even at the inflection point, the predicted values are consistent with the measured values, which can accurately reflect the data mutation. The RMSE of the proposed model is 0.98, MAPE is 0.34, and MAE is 0.41. The experimental result indicates that the proposed model is an effective method for predicting structural deformation. So it can be better applied to engineering deformation prediction to ensure the safety during the structure construction and operation stages.

### 4.3. Experimental Performance Analysis

In order to further evaluate the performance of the proposed model, the proposed model is compared with the wavelet neural network (WNN), deep belief networks and support vector regression (DBN-SVR), gated recurrent unit (GRU), and LSTM models in this study. The hyperparameters are critical to the prediction performances, and the cross-validation of multiple tests yields the optimal hyperparameter values for different models. The specific hyperparameters are as follows:

  WNN: in the wavelet neural network, the number of hidden layer neurons is 3, the number of training is set as 5000, and the kernel function is radial basis function (RBF kernel)
  DBN-SVR: the number of network layers of the DBN model is set as 3. The temporal characteristics are extracted through the two stages of pretraining and fine-tuning of the DBN network. Finally, the output is predicted by SVR. The kernel function of the SVR is RBF, the time of training is set as 10000, and the penalty factor is 0.01.
  LSTM: the number of network layers in LSTM is 3, and the number of neurons in each layer is set as 24. Each layer is followed by regularization to prevent overfitting. The batch size is 16.
  GRU: the number of network layers in the GRU is 3, and the number of neurons in each layer is set as 24. Each layer is followed by a regularization to prevent overfitting, and the batch size is 8.

**Figure 9:**
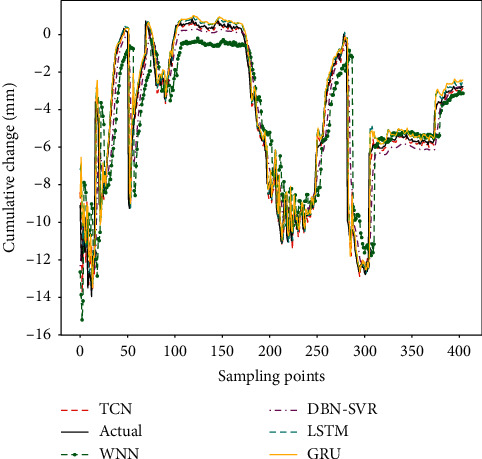
The prediction results with different models.

The prediction results of different models are shown in Figure 8, and performances analysis results are shown in Figure 9.

**Figure 10:**
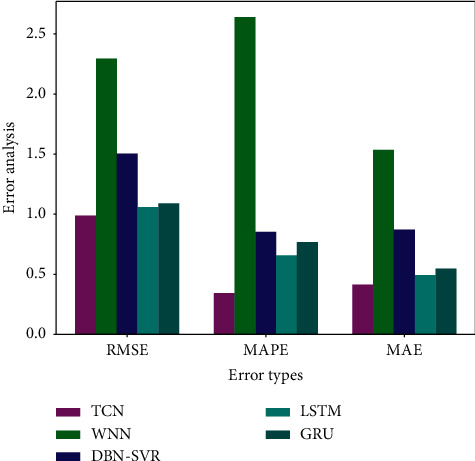
The error analysis for different models.

From Figures 9 and 10, it can be seen that the prediction result of the WNN is the worst. The overall prediction values can reflect the trend of data change, but the local difference is very large. The performance of DBN-SVR is better than the WNN. DBN can map the original data into the high-level feature space from the bottom layer after the layer-by-layer unsupervised training, and the model parameters are adjusted by supervised reverse trimming, which can effectively improve the prediction performance. Both the LSTM and GRU models are gated-based recurrent neural networks that retain the information needed by various gates as much as possible and can better capture the dependence by selectively forgetting unwanted information in the time series. Although LSTM and GRU models also achieved better prediction results, they are also worse than the TCN model. The predicted value of the TCN model is the closest to the real value, which can accurately reflect the change trend of structural deformation. In the TCN model, by stacking the convolutional layer, increasing the dilated factor, and enlarging the size of the convolution kernel, the receptive field is also increased, so that the memory length of the model is better controlled. This avoids the gradient explosion or gradient disappearance that often occurs in RNNs due to the difference in back propagation path and sequence time direction. Due to the large-scale parallel processing in the TCN, the parameters of the training are less, which greatly reduces the time of network training. At the same time, the residual connection can effectively improve the model accuracy.

In order to compare the specific errors values in different prediction methods, performance comparison results for different models are given in Table 4.

According to Table 4, the various error values for the proposed model are the minimum compared with WNN, DBN-SVR, GRU, and LSTM models. The RMSE of the TCN model, respectively, decreases 56.95%, 34.33%, 9.4%, and 6.8%, and the average RMSE is reduced by 26.88%. The MAPE of the TCN model, respectively, decreases 86.97%, 59.71%, 55.28%, and 46.67%, and the average MAPE is reduced by 62.16%. The MAE of the TCN model, respectively, decreases 70.04%, 52.67%, 24.41%, and 16.18%, and the average MAE is reduced by 40.83%. The prediction results show that the proposed model has a smaller error than traditional models, and it is an effective prediction model for structural deformation.

## 5. Conclusions

In view of the insufficient feature extraction of traditional time prediction models, this study proposes a structural deformation prediction model based on the TCN. Since the structural deformation data have temporal correlation characteristic, first, the temporal features can be extracted by the TCN model, the long-term memory of the time series is obtained by dilated convolution, and the causal convolution is realized by padding to prevent information leakage effectively. The residual connection can reduce the model prediction error. Second, the predicted output can be obtained through full connection. Finally, the hyperparameters of the model are optimized by the orthogonal experiment, and the optimized parameter combination is selected as the parameter of the model, which can fully extract features from the deformation data. The prediction results with the optimal parameters are obtained through the full connection network. Experimental results indicate that the TCN has smaller prediction error when dealing with structural deformation time series. The prediction performances of the proposed model are proved by comparing with WNN, DBN-SVR, LSTM, and GRU. The TCN model is able to extract temporal features with the increase of TCN layers number, which will make it difficult to train the model and prolong the running time. On the other hand, the multiple sensors are generally used for the same measuring point for the deformation monitoring in the structural engineering. The monitoring data of the multiple sensors usually have a certain relationship in temporal and spatial characteristics. We will further explore the spatial characteristics of the monitoring data to improve the accuracy of the prediction model in the future.

## Figures and Tables

**Table 1:** Types and levels of orthogonal experimental factors.

	Types of factors (*θ*)	Levels			
		1	2	3	4
A	Kernel size	5	6	7	8
B	Kernel numbers	8	16	24	32
C	Dilation factor	8	16	32	64
D	TCN layer number	8	12	16	20
E	Learning rate	0.0001	0.001	0.01	0.05

**Table 2:** Experimental environment.

CPU	Intel (R) Core (TM) i5-6200U @2.30 GHZ
RAM	4 GB
Operating system	Windows (64)
Python	3.7

**Table 3:** Orthogonal experiment results.

Test number	Types and levels of factors					RMSE	MAPE	MAE	Running time (min)
	A	B	C	D	E				
1	5	8	8	8	0.0001	1.08	1.13	0.66	7.73
2	5	16	16	12	0.001	1.05	0.86	0.53	38.73
3	5	24	32	16	0.01	2.26	5.61	1.70	97.90
4	5	32	64	20	0.05	9.09	9.93	7.26	247.30
5	6	8	16	16	0.05	1.08	1.07	0.58	45.34
6	6	16	8	20	0.01	1.10	0.84	0.57	47.70
7	6	24	64	8	0.001	1.05	0.64	0.49	75.61
8	6	32	32	12	0.0001	2.41	1.83	1.69	87.78
9	7	8	32	20	0.001	1.22	0.63	0.47	77.55
10	7	16	64	16	0.0001	1.20	1.47	0.74	129.25
11	7	24	8	12	0.05	1.17	1.73	0.74	36.68
12	7	32	16	8	0.01	1.08	0.76	0.51	39.23
13	8	8	64	12	0.01	1.08	0.89	0.57	68.86
**14**	**8**	**16**	**32**	**8**	**0.05**	**0.98**	**0.34**	**0.41**	**41.04**
15	8	24	16	20	0.0001	1.18	1.5648	0.73	98.53
16	8	32	8	16	0.001	1.28	1.3177	0.83	63.74

The bold values represent the best prediction result of the model when the TCN model takes this set of parameters.

**Table 4:** The performance comparison for different models.

Models	RMSE	MAPE	MAE
WNN	2.2940	2.6395	1.5349
DBN-SVR	1.5038	0.8533	0.8724
GRU	1.0901	0.7688	0.5474
LSTM	1.0600	0.6570	0.4937
TCN	0.9876	0.3438	0.4138

## Data Availability

The data used to support the findings of this study are available from the corresponding author upon request.
